# A new IDH-independent hypermethylation phenotype is associated with astrocyte-like cell state in glioblastoma

**DOI:** 10.1186/s13059-025-03670-y

**Published:** 2025-07-03

**Authors:** Ana Luisa Costa, Daria Doncevic, Yonghe Wu, Lin Yang, Ka Hou Man, Anna-Sophie Spreng, Hannah Winter, Michael Nai Chung Fletcher, Bernhard Radlwimmer, Carl Herrmann

**Affiliations:** 1 Institute for Pharmacy and Molecular Biotechnology (IPMB) and BioQuant, Heidelberg University, Heidelberg, 69120 Germany; 2 Department of Pathology, Institute of Basic Medical Sciences Chinese Academy of Medical Science, School of Basic Medicine Peking Union Medical College; Molecular Pathology Research Center, Chinese Academy of Medical Sciences, Beĳing, 100730 China; 3 Division of Molecular Genetics, German Cancer Research Center (DKFZ), Heidelberg, 69120 Germany

## Abstract

**Background:**

DNA methylation plays a crucial role in cancer development and progression and has been linked to genetically and clinically distinct tumor classes, including IDH-mutated and IDH-wildtype adult-type diffuse gliomas. Here, we identify a CpG-island methylator phenotype (CIMP) that characterizes the receptor tyrosine kinase 2 (RTK2) subtype of IDH-wildtype glioblastoma.

**Results:**

This RTK2-CIMP affects genomic locations and cell functions distinct from those of IDH mutation-associated IDH-CIMP and suppresses the expression of its target genes. The RTK2-CIMP-region chromatin is characterized by a combination of repressive and activating marks, including polycomb-associated H3K27me3 and enhancer-associated H3K4me1, consistent with DNA methylation-mediated silencing of genes with bivalent-state promoters in neural progenitor cells. Functionally, RTK2-CIMP affects neuronal lineage genes and is significantly associated with astrocyte-like glioblastoma, suggesting that RTK2-CIMP is an epigenetic signature of the astrocyte-like cell state. Furthermore, we demonstrate that RTK2-CIMP can be induced by genetic manipulation in glioblastoma cells.

**Conclusions:**

Our results suggest that RTK2-CIMP is a key contributor to cell-state plasticity in glioblastoma.

**Supplementary Information:**

The online version contains supplementary material available at 10.1186/s13059-025-03670-y.

## Background

Epigenetic regulation is essential for cell reprogramming, fate commitment, tissue homeostasis, and differentiation. In cancer, alterations in these epigenetic mechanisms are associated with increased proliferation, dedifferentiation, and tumor progression [1]. While somatic mutations often drive tumor initiation, genetic mechanisms are frequently complemented or substituted by epigenetic aberrations that contribute to tumor progression [2]. DNA methylation is one of the key epigenetic modifications involved in coordinating the transcription and repression of genes, silencing of transposable elements, modulating interactions between DNA and transcription factors (TFs), and recruiting chromatin modifiers [3, 4]. One of the most commonly observed DNA methylation aberrations in cancer is the hypermethylation of CpG islands (CGIs) [5]. Located in the promoter region of approximately half of all protein-coding genes, CGIs are GC-rich regions that are normally unmethylated in healthy tissues, except in areas of the genome where methylation is essential for gene repression, such as imprinted loci and the inactive X chromosome in female mammalian cells [6–8]. Hypermethylation of CGIs at the promoters of tumor suppressor genes is considered to be a key driver of carcinogenesis, and accordingly, therapeutic agents that target the responsible epigenetic enzymes have also shown some efficacy in specific tumors [9–11].

Concurrent hypermethylation of large numbers of CpG islands, commonly referred to as CGI methylator phenotype (CIMP), has been described in colorectal cancer, gliomas (G-CIMP), gastric cancer, breast cancer, and acute myeloid leukemia (AML), among other cancer types [12–21]. In both tumors and healthy cells, DNA methylation involves complex interactions with other epigenetic modifications that also play roles in gene regulation, such as monomethylation at lysine 4 of histone H3 (H3K4me1), trimethylation at lysine 36 of histone H3 (H3K36me3), or Polycomb-dependent modification H3K27me3 [22–24]. Within CGIs, aberrant DNA methylation is known to be related to specific patterns of H3K4me3, H3K9me3, and H3K27me3 modifications [25–27]. Typically, DNA methylation and H3K4me2/3 activation are mutually exclusive, while interplay with H3K27me3 is much more complex and context dependent, as DNA methylation and H3K27me3 can either act together to repress gene expression or antagonistically [28, 29]. CGIs at the promoters of developmental genes occupied by Polycomb repressor complexes in healthy adult tissue encompass a large percentage (approximately 75%) of hypermethylation-prone CGIs in cancer, although the molecular mechanisms underlying this hypermethylation and the determinants of tumor- and cell type-specificity remain unclear [26, 30, 31].

One of the tumor types in which aberrations in DNA methylation, and particularly CIMP, have been extensively described is glioblastoma, an aggressive brain tumor with poor prognosis that accounts for 14% of all tumors and 50% of all malignant brain tumors [32, 33]. Glioblastoma can be categorized into 4 molecular subtypes with distinct DNA methylation and transcriptomes: IDH, MES (mesenchymal), RTK1 (receptor tyrosine kinase 1), and RTK2 [34–36]. RTK2 tumors are characterized by the enrichment of recurrent chromosomal alterations, including gain of chromosome 7 with or without EGFR amplification, loss of 9p21 (CDKN2A/B), and chromosome 10 loss. Gain of chromosome 19 and 20 is also recurrently observed (40% of cases). The RTK2 glioblastoma subtype aligns with the RTK2 subtype of Capper et al. and overlaps the TCGA Classical gene expression and LGM4 DNA-methylation subgroups [34–36]. Extent of resection and MGMT promoter methylation have been proposed as independent prognostic factors [37, 38].

CIMP was previously reported in the IDH subtype, where it is caused by mutations in isocitrate dehydrogenase (*IDH*) metabolic enzymes, IDH1 in the cytoplasm, and IDH2 in the mitochondria [39, 40]. *IDH1/2* mutations are also proposed to be the origin of CIMP in other tumors, such as AML or chondrosarcoma [41]. Although phenotype is often associated with specific tumor molecular subtypes, various causal events and associations with tumor features and patient survival have been identified across other tumor types (Karpinski et al. 2018; Saghafinia et al. 2018; Dahlin et al. 2010). An IDH-independent “alternative” CIMP has been described in AML and is associated with better overall survival [42]. In colorectal cancer, CIMP is linked to the BRAF^V600E^ mutation and to microsatellite instability [43], while another alternative CIMP, driven by KRAS mutation(+), has been reported in colorectal cancer and linked to adverse patient prognosis [43–45].

In this work, we have revisited the CIMP phenomenon in glioblastoma using a comprehensive dataset of 60 whole-genome bisulfite sequencing datasets encompassing the four major DNA methylation subtypes [46]. We identified an alternative, IDH-independent CIMP phenomenon that manifests in the RTK2 subtype (RTK2-CIMP) and showed that it displays different functional and epigenetic characteristics than IDH-CIMP, in particular a tight association with regions marked by Polycomb-associated H3K27me3. Additionally, we show that the regions affected by either type of CIMP can be predicted using a random forest classifier based on epigenetic data from healthy early progenitor cells, suggesting that DNA hypermethylation propensity is already encoded in normal progenitor cells before malignant transformation. We found epigenetic commonalities with AML alternative CIMP, suggesting that Polycomb-related alternative CIMP might be involved in the general process of malignant transformation. Finally, by analyzing single-cell transcriptomic data from healthy progenitor and tumor cells, we demonstrated that RTK2-CIMP specifically affects genes involved in neuronal differentiation and contributes to the repression of specific cell states, making it a modulating element of the cell state and tumor composition in glioblastoma.

## Results

### Glioblastoma tumors exhibit two distinct types of CpG island DNA hypermethylation

**Fig. 1:**
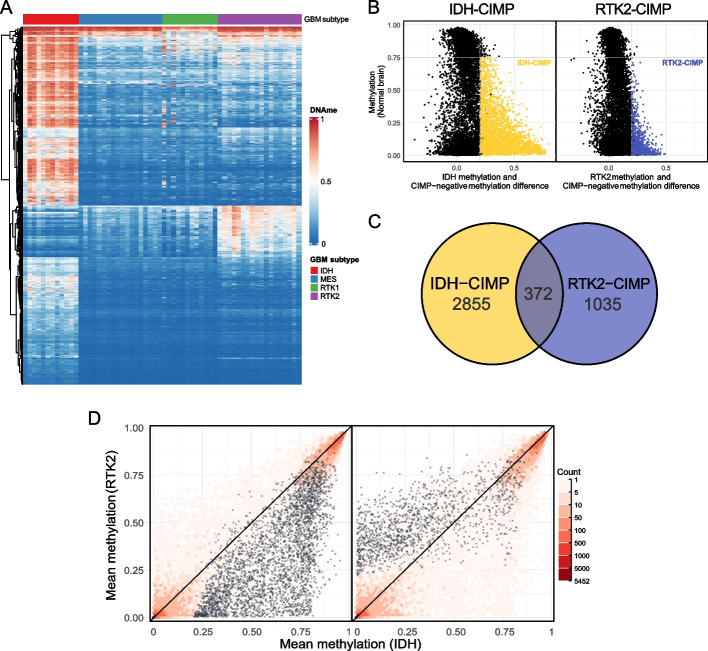
A unique RTK2-related hypermethylation phenotype is distinct from CIMP caused by IDH mutations. **A** The top 2000 most significant CpGs were selected using ANOVA testing between subtypes showing the patterns of subtype-specific methylation. **B** Scheme representing the thresholds used for the definition of CIMP in IDH-CIMP (left, in yellow) and RTK2-CIMP (right, in blue) subtypes. Each dot represents one CGI, with black dots representing non-CIMP CGIs. Minimal levels of methylation in the normal brain (horizontal) and the minimal difference between the CIMP-positive (IDH or RTK2) and CIMP-negative subtypes (MES and RTK1) are represented. **C** Size of the RTK2-CIMP and IDH-CIMP groups and of their overlap. **D** Mean methylation of IDH (left) and RTK2-CIMP (right) CGIs in comparison with the methylation distribution of all CGIs (red hexagon bins) in IDH and RTK2 samples, respectively. The hexagon bin color represents the number of CGIs. CIMP-CGIs are represented as black crosses

To survey CIMP in glioblastoma, we analyzed whole-genome DNA methylation data [46] comprising 60 glioblastoma samples of the four major DNA methylation subtypes: IDH (*n* = 12), MES (*n* = 18), RTK1 (*n* = 12), and RTK2 (*n* = 18). We focused our DNA methylation analysis specifically on CpG islands (CGIs, as defined by the UCSC Genome Browser) and calculated the average methylation beta values of CpGs within a total of 26,268 CGIs. These CGIs displayed a global increase in DNA methylation in glioblastoma compared to normal brain tissue. Within glioblastoma, CGI median methylation was highest in IDH samples and lowest in RTK1 samples (Additional File 1: Fig S1A), consistent with reported global hypomethylation of RTK1 [46]. Unsupervised analysis of the DNA methylation levels of the 2000 most significant CGIs according to an ANOVA test between subtypes revealed the known DNA hypermethylation of a large set of CGIs in IDH-mutated tumors. However, this analysis also highlighted a set of CGIs showing relative DNA hypermethylation specifically in the RTK2 samples (Fig. 1A). Next, we defined 3227 IDH and 1407 RTK2 CGIs that met the criteria for CIMP defined by Issa 2004: (i) hypermethylation relative to the CIMP-negative MES and RTK tumors; abs (methylation beta difference) > 0.2), (ii) lower methylation in normal brain (methylation beta < 0.75), and (iii) statistical significance in subtype-specific testing (Kruskal‒Wallis test, adj. *p* < 0.001) (Fig. 1B, C and Additional File 1: Fig S1B). We will refer to these hypermethylated CGIs collectively as IDH-CIMP-CGIs and RTK2-CIMP-CGIs, respectively.

Genome-wide CGI methylation was strongly correlated between IDH and RTK2 tumors (Fig. 1D, red dots), but IDH-CIMP and RTK2-CIMP displayed subtype-specific hypermethylation (Fig. 1D, black crosses). Interestingly, the average methylation over the RTK2-CIMP CGI regions in all samples is not associated with the EGFR amplification status (Additional File 1: Fig S1F). The RTK2-CIMP-CGIs tended to have more CpG dinucleotides than IDH-CIMP-CGIs (88 ± 82 vs 66 ± 59 CpGs, Additional File 1: Fig S1C) and showed enrichment in intergenic regions (26.5% vs 17.3%) compared to the IDH-CIMP (one-sided binomial test, *p* < 2.2e − 16) (Additional File 1: Fig S1D). Furthermore, the expression of genes with IDH-CIMP-CGIs or RTK2-CIMP-CGIs in their promoter or intragenic regions (Additional File 1: Fig S1E) was lower than that of genes harboring non-CIMP CGIs, suggesting that CIMP indeed suppresses gene expression (Additional File 1: Fig S1G).

To validate RTK2-CIMP in an external glioblastoma cohort, we annotated *N*=131 IDH-wildtype glioblastoma samples from The Cancer Genome Atlas (TCGA) cohort as MES, RTK1, and RTK2 subtypes based on DNA methylation, as described previously [37]. In the TCGA cohort, RTK2-CIMP-CGIs also displayed subtype-specific DNA hypermethylation in RTK2 tumors (Additional File 1: Fig S2A, B). We also defined de novo RTK2-CIMP CGI regions in the TCGA RTK2 samples and found 1163 such regions, of which 556 overlap the RTK2-CIMP CGI defined in our GBM cohort (Additional File 1: Fig S2D). To verify whether RTK2-CIMP could also be detected at single-cell resolution, we used a published resource that includes matched DNA methylation and transcriptomics data from glioblastoma [47]. Based on cell fractions, RNA expression and DNA amplification status, we defined distinct sets of 3 samples representing the RTK2 subtype and non-RTK2 subtype (see Methods). Similarly, RTK2-CIMP-CGI methylation was higher in RTK2 tumors than in non-RTK2 tumors, whereas background methylation at non-RTK2-CIMP-CGIs did not differ (Additional File 1: Fig S2C). Thus, we could validate specific DNA hypermethylation at RTK2-CIMP-CGIs in RTK2 tumors in two independent datasets, strongly suggesting that this hypermethylation is a general feature of the RTK2 glioblastoma subtype.

### IDH-CIMP and RTK2-CIMP correspond to distinct chromatin signatures

Given the crosstalk between DNA methylation and other epigenetic features [25, 48–50], we assessed how IDH-CIMP and RTK2-CIMP are related to histone modifications. We analyzed a matched dataset of 6 histone modifications in the four glioblastoma subtypes, including the activating marks H3K27ac, H3K4me1, and H3K4me3; the repressive marks H3K27me3 and H3K9me3; and the gene body/transcription-associated mark H3K36me3, as published in our past work [46]. To determine whether CGIs affected by DNA hypermethylation in glioblastoma have distinct epigenomic signatures during normal brain development, we included the corresponding histone modifications and DNA methylation in neural progenitor cells (H9-derived neural progenitor cells, NPs) [51, 52]. NPs are the healthy cells that most resemble glioblastoma stem cells, making them good counterparts for comparative analysis [53]. Our goal here is to determine whether the chromatin context, as defined by the combination of histone modifications and DNA methylation, is specific for RTK2-CIMP CGI regions, and possibly different from the chromatin state of IDH-CIMP CGI regions.

**Fig. 2:**
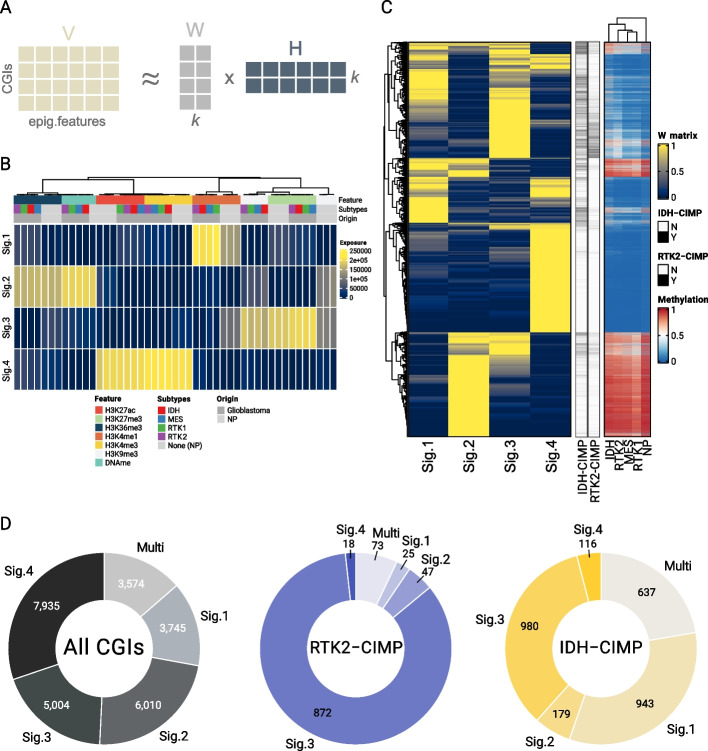
CGIs are associated with distinct chromatin signatures. **A** Illustration of the NMF-based decomposition applied to all CGIs (rows of V) characterized by their respective features (columns of V). Using k (signatures), the product of W and H is approximately equal to V and can be used to reconstruct it. **B** Heatmap representation of the H matrix exposure values by epigenetic modification (as columns) for each NMF-derived signature (as rows). The color indicates the exposure value of a given feature to each signature. Both glioblastoma (in dark gray) and NP (in light gray) samples are shown. **C** Heatmap representation of the W matrix values according to the NMF-derived signature across all CGIs (left) and comparison of the W matrix values with the DNA methylation levels in the glioblastoma subtypes and in the NPs. Higher values (yellow) on the W matrix heatmap indicate that one CGI is more likely to be associated with one signature. RTK2- and IDH-CIMP are also represented in black on the middle annotation bar. **D** Fractions and numbers of RTK2-CIMP (blue tones) and IDH-CIMP (yellow tones) CGIs associated with each signature or assigned to more than one signature. The global assignment fractions of all CGIs are shown on the left (in gray tones)

We applied nonnegative matrix factorization (NMF) to identify distinct chromatin signatures defined by combinations of DNA methylation and histone modifications and assign the CGIs to these signatures (Fig. 2A–C) [54]. This approach is similar to the one used, e.g., to define mutational signatures in cancer samples [55]. For each CGI region, we determined the signal of the histone marks and DNA methylation over this region and summarized this into a matrix consisting of epigenetic features as columns and CGIs as rows. NMF aims to decompose this matrix into one matrix W with signatures as columns and CGIs as rows and H with epigenetic features as columns and signatures as rows (Fig. 2A). The values in these two matrices can be interpreted as the contribution of each epigenetic feature to the definition of the signature (W matrix, Fig. 2B) or the importance (or exposure) of each chromatin signature for each CGI region (H matrix, Fig. 2C). Using this approach, we identified four chromatin signatures characterized by different combinations of activating/repressing modifications and variable contributions from NPs or tumor samples (Fig. 2B, Additional File 1: Fig S3A). Next, we assigned each of the 26,268 CGIs to one or multiple of the four signatures using their NMF exposures, i.e., the values in the H matrix (Fig. 2C,D; Sig.1 = 14.3%, Sig.2 = 22.9%, Sig.3 = 19.0%, Sig.4 = 30.2%, and 13.6% multiply assigned) (see *Methods*). To verify whether the subset of CGIs affected by CIMP was associated with specific signatures, we assessed the distribution of the subset of IDH-CIMP-CGIs and RTK2-CIMP-CGIs compared to all CGIs (Fig. 2D). The analysis showed that while only 37% of the IDH-CIMP-CGIs were associated with Signature 3, this was the case for 77% of the RTK2-CIMP-CGIs (*log2* odds ratio = 2.14). Signature 3 was highly enriched for the polycomb-associated repressive H3K27me3 mark and the active enhancer mark H3K4me1 in NPs, suggesting that it corresponds to a bivalent H3K4me1/H3K27me3 state in progenitor cells (Fig. 2B, Additional File 1: Fig S3A). In contrast, IDH-CIMP showed the strongest enrichment for signature 1, characterized as an active state enriched in H3K4me1 (*log2* odds ratio = 1.21 versus − 2.56). In summary, IDH-CIMP appears to be associated with multiple chromatin signatures, while RTK2-CIMP shows a strong association with bivalent signature 3.

### Progenitor chromatin states are predictive of future hypermethylation classes

**Fig. 3:**
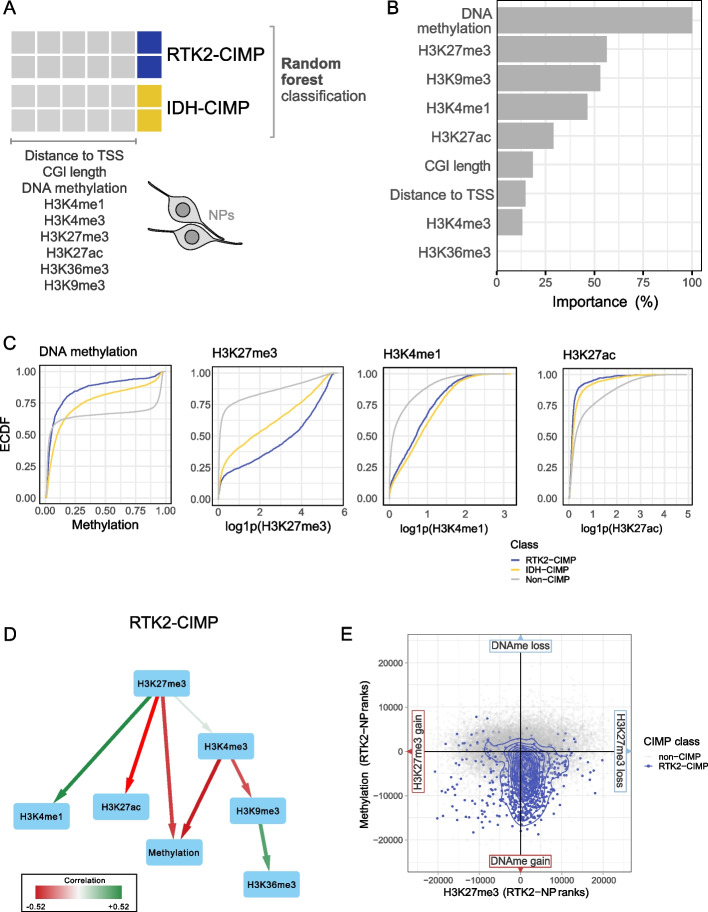
CIMP occurrence is predetermined in progenitor cells. **A** Diagram of the RF classifier used for distinguishing between IDH-CIMP and RTK2-CIMP in healthy cells using NP epigenomic data (DNA methylation and 6 histone modifications) together with 2 other CGI-specific features (CGI length and distance to the TSS). **B** Feature importance (as a percentage, on the *x*-axis) obtained from the RF classification model after training to distinguish IDH-CIMP from RTK2-CIMP. **C** Empirical distribution function curve for DNA methylation of various histone marks by CGI group (color-coded for non-CIMP CGIs, RTK2-CIMP, and IDH-CIMP) on the NP. **D** Bayesian networks inferred from RTK2-CIMP-CGIs representing the direction of influence between the epigenetic components. Edge color represents a positive (green) or negative (red) correlation, and edge thickness represents the strength of the inferred relation after model training. **E** DNAme gain/loss vs. H3K27me3 gain/loss comparing the RTK2 signal to the NP signal in non-CIMP CGIs (gray dots) and RTK2-CIMP-CGIs (blue dots)

Next, we trained a random forest classifier on NP chromatin data to identify CGI features in progenitor cells that are predictive of tumor cell IDH-CIMP-CGIs and RTK2-CIMP-CGIs (Fig. 3A). The classifier distinguished the two classes of CIMP regions with an AUC of 0.796, indicating that the chromatin context of the two types of CIMP already differs in NPs prior to tumorigenesis. In the classifier, DNA methylation had the highest feature importance, indicating that DNA methylation in NPs clearly distinguished the two sets of affected CGIs (Fig. 3B). We confirmed the importance of these features by examining their distribution across the different CIMP regions. Both IDH-CIMP-CGIs and RTK2-CIMP-CGIs were equally enriched for the activating marker H3K4me1 compared to non-CIMP-CGIs (Fig. 3C). However, RTK2-CIMP-CGIs were significantly more strongly enriched for H3K27me3 compared to IDH-CIMP-CGIs. These observations are consistent with our hypothesis that RTK2-CIMP-CGIs are characterized by a combination of repressive and activating marks, defining a bivalent progenitor state in NPs. We further confirmed this by training two alternative RF classifiers to distinguish (1) IDH-CIMP-CGIs from non-CIMP-CGIs and (2) RTK2-CIMP-CGIs from non-CIMP-CGIs. Both analyses indicated the specific association of these two sets of CIMP-CGIs with distinct chromatin states in the NPs (Additional File 1: Fig S4A, B).

To understand the dynamic associations between histone modifications and DNA methylation at RTK2-CIMP-CGIs, we generated a directed network representation through Bayesian network modeling of the NPs (see Methods). Bayesian networks have been used to describe the direction of influence between factors, for example, between various epigenetic components [56]. Here, we inferred the most likely structure of the influence graph between epigenetic components using the data observed in RTK2-CIMP-CGIs. Using a bootstrapping approach, we obtained a network representing the predicted influence between epigenetic modifications, represented as directed interactions (Fig. 3D). The network highlighted the central importance of H3K27me3 in the NPs and its direct connection to the activating histone mark H3K4me1. Strikingly, the correlation between H3K27me3 and H3K4me1 (indicated by the color of the edge) was positive, indicating the coexistence of these marks in NPs, as expected in bivalent regions. Furthermore, to visualize the concurrent changes in H3K27me3 and DNA methylation between NP cells and RTK2 tumors, we plotted the respective rank differences (rank in tumor minus rank in NP, see Methods) for both marks (Fig. 3E). Remarkably, RTK2-CIMP-CGIs mapped to the DNA methylation gain and H3K27me3 loss quadrant of the plot, whereas non-CIMP CGIs were distributed around the plot center. This analysis suggested that the loss of NP H3K27me3 is compensated for by DNA hypermethylation in RTK2 glioblastoma cells, maintaining the repression of RTK2-CIMP regions.

### RTK2-CIMP targets neuronal development genes

**Fig. 4:**
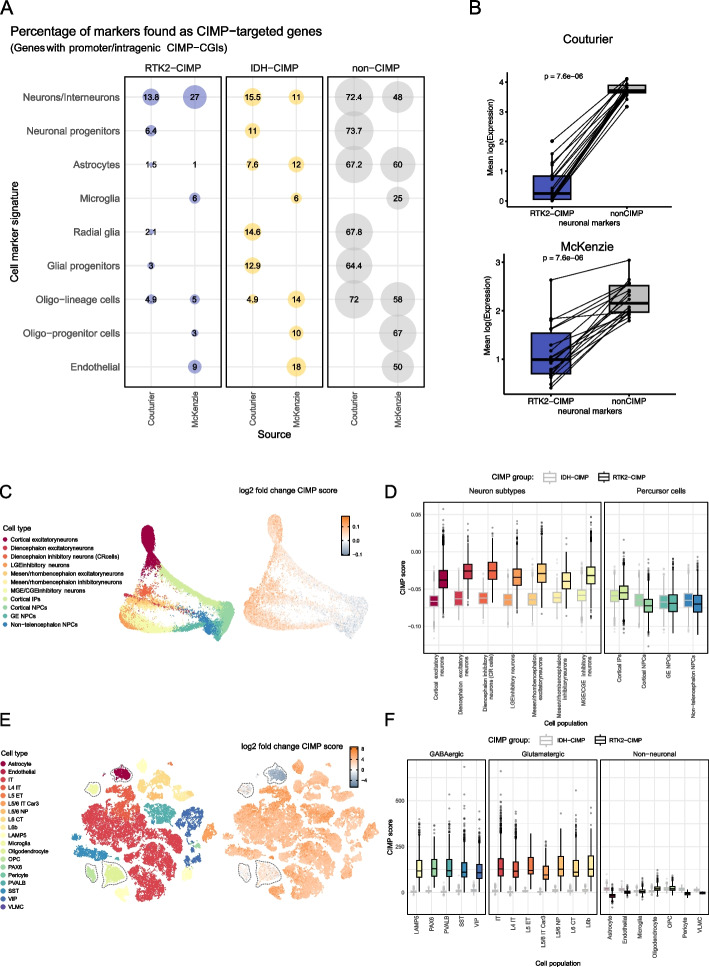
CIMP target genes are associated with neuronal function and differentiation. **A** Percentage of cell markers from the two distinct source datasets [61, 62] targeted by RTK2-CIMP (in blue) and IDH-CIMP (in yellow). The percentages are represented as both numbers and dot sizes. The CIMP groups were compared with the remaining non-CIMP CGI-genes (in gray). Cell markers were sometimes collapsed to ease visualization. **B** Expression of the neuronal markers in the GBM cohort, stratified by genes that are targets of a RTK2-CIMP CGI or not. Each dot represents one RTK2 sample, and the *y*-value represents the average expression of the neuronal markers which are RTK2-CIMP CGI targets (left, blue box) or no (right, grey box). The lines connect the identical samples. Top: Couturier marker gene set; Bottom: Mc Kenzie marker gene set. **C** SPRING reconstruction of the cell populations in the developing brain (organoid) dataset [63] (*left*). The cell annotations are indicated in the legend on the left. Log2 fold change in the RTK2-CIMP expression score compared with the IDH-CIMP expression score obtained for the same cells is shown on the right. The log2-fold changes are labeled on the continuous color scale on the leftmost side. **D** Distribution of the CIMP expression scores of the RTK2-CIMP (black outline) and IDH-CIMP (gray outline) groups by cell population (as represented in B). Cells are faceted by their identity as neuron-like or precursor-like types. **E** t-SNE representation of the cell populations in the adult brain dataset [64] (*left*). Log2 fold change in the RTK2-CIMP expression score compared with the IDH-CIMP expression score obtained for each cell is also shown (*right*). Log2 fold change values are labeled on the continuous color scale on the leftmost side. Non-neuronal cell clusters are highlighted. **F** CIMP expression scores of the RTK2-CIMP (black outline) and IDH-CIMP (gray outline) groups according to cell population (as represented in C). Cells are faceted as two groups of neuron subtypes (GABAergic or glutamatergic) or non-neuronal group

The temporal control of normal neurodevelopment is precisely coordinated by various factors, including dynamic DNA demethylation [57–60]. To determine whether the RTK2-CIMP genes are associated with particular aspects of neurodevelopment, we intersected the RTK2-CIMP genes and gene signatures of brain cell types [61, 62]. We found a high prevalence of neuronal markers among the RTK2-CIMP genes. In contrast, IDH-CIMP genes were distributed across all cell-type marker sets and were comparable to non-CIMP-associated genes (Fig. 4A). This finding suggested that RTK2-CIMP specifically targets genes involved in neuronal differentiation and leads to their silencing through hypermethylation. To verify if the RTK2-CIMP hypermethylation leads to a silencing of these genes, we stratified the neuronal markers into those targeted by an RTK2-CIMP CGI (RTK2-CIMP) and those not associated with an RTK2-CIMP CGI (non CIMP). We computed the average expression of these two sets for each RTK2 sample, performing the analysis separately for the Couturier set and the McKenzie set. We observe a strong downregulation of the expression of the neuronal markers which are RTK2-CIMP targets, compared to the non-CIMP set, for both sets of markers, indicating that the hypermethylation leads to a strong silencing of these markers (Fig. 4B). At the gene level, we observe a strong negative correlation between methylation of the associated CGI and expression level of the neuronal markers for both sets of marker genes (Additional File 1: Fig S5). Interestingly, this correlation is stronger (i.e., more negative) when computed in the RTK2 samples, compared to non-RTK2 (i.e., MES and RTK1) samples, indicating a more direct relationship between hypermethylation and repression in RTK2 samples. This increased negative correlation is statistically significant for both sets of marker genes (Additional File 1: Fig S5).

Having shown that the hypermethylation leads to a silencing of neuronal marker genes, we hypothesized that the RTK2-CIMP hypermethylation might suppress the normal neuronal differentiation process. Hence, we checked the expression of the neuronal markers associated with an RTK2-CIMP CGI, over the course of neural development using a human brain development dataset [63] (Fig. 4C, D). To evaluate the potential impact of gene silencing through RTK2-CIMP hypermethylation, we defined the RTK2-CIMP expression score for each cell as the average expression of genes targeted by an RTK2-CIMP-CGI. Hence, nonmalignant cells with a high RTK2-CIMP expression score express the genes targeted by RTK2-CIMP at a higher level, increasing their susceptibility to RTK2-CIMP in the malignant context. Each cell was assigned an RTK2-CIMP expression score, while IDH-CIMP expression scores defined using genes targeted by IDH-CIMP-CGIs were used for comparison. We found that RTK2-CIMP expression scores were higher in differentiated cells of the neuronal lineage than in precursor cells (one-sided Wilcoxon signed rank test, *p* < 2.2e − 16), consistent with the hypothesis that RTK2-CIMP suppresses neuronal differentiation (Fig. 4C). In contrast, IDH-CIMP expression score was not associated with any particular cell lineage or stage (Fig. 4D). As this dataset covered only neuronal differentiation, we repeated the analysis in an independent adult brain cell dataset and showed that RTK2-CIMP expression scores were much higher in adult neurons (one-sided Wilcoxon signed rank test, *p* < 2.2e − 16), both excitatory and inhibitory than in nonneuronal cells (Fig. 4E, F). These findings confirmed that RTK2-CIMP specifically affects neuronal lineage cells.

In a recent publication, Drexler et al. described subclasses of IDH-wt glioblastoma with high or low neural characteristics [65]. They identified 1289 differentially methylated CpGs between these two classes, with 255 hypermethylated in high-neural (high-neural CpGs) and 1034 hypermethylated in low-neural (low-neural CpGs), consistent with our observation that the hypermethylation is linked to the repression of the neural program. The 255 high-neural CpGs can be mapped to 32 CGIs, whereas the 1034 low-neural CpGs can be associated with 72 CGIs. Comparing these CGIs with the RTK2-CIMP-CGIs identified previously, 9 of the 72 CGIs associated with low-neural glioblastoma overlap with the RTK2-CIMP CGIs, whereas none of the high-neural CGIs overlaps. While this overlap is modest, this analysis is consistent with the fact that the RTK2-CIMP CGIs identified in our study are associated with a repression of neuronal markers.

To investigate if the RTK2-CIMP phenotype is associated to clinical differences, we returned to the RTK2 glioblastoma samples of the TCGA cohort, and computed for each RTK2 sample an RTK2-CIMP methylation score based on the level of hypermethylation of the RTK2-CIMP CGIs. We then performed a Cox-proportional hazard analysis, and found a significant association between stronger hypermethylation and increased survival (*n* = 58, Coefficient = − 4.51, *p*-value = 0.0245, see Methods). This again is consistent with the observation in [65] showing that low-neural glioblastoma have a better survival probability compared to high-neural.

### RTK2-CIMP is linked to glioblastoma cell state heterogeneity

Next, we aimed to determine how RTK2-CIMP affects cellular heterogeneity in glioblastoma. In this context, it is essential to emphasize that DNA hypermethylation in tumors might modulate lineage differentiation programs that are similar but not identical to those of normal neurodevelopment. Thus, tumor cells remain progenitor-like or differentiated-cell-like malignant cells despite partial activation or suppression of gene expression signatures associated with cell type differentiation in normal tissues.

Recent single-cell analysis of the intratumoral heterogeneity of IDH-wild-type tumors recently led to a new model of four cellular states in glioblastoma: (i) oligodendrocyte-progenitor-like (OPC-like), (ii) neural-progenitor-like (NPC-like), (iii) astrocyte-like (AC-like), and (iv) mesenchymal-like (MES-like) states. These cell states have been linked to specific somatic alterations, such as amplification of *CDK4* (NPC-like), *EGFR* (AC-like), and *PDGFRA* (OPC-like) and mutation of *NF1* (MES-like) [66].

**Fig. 5:**
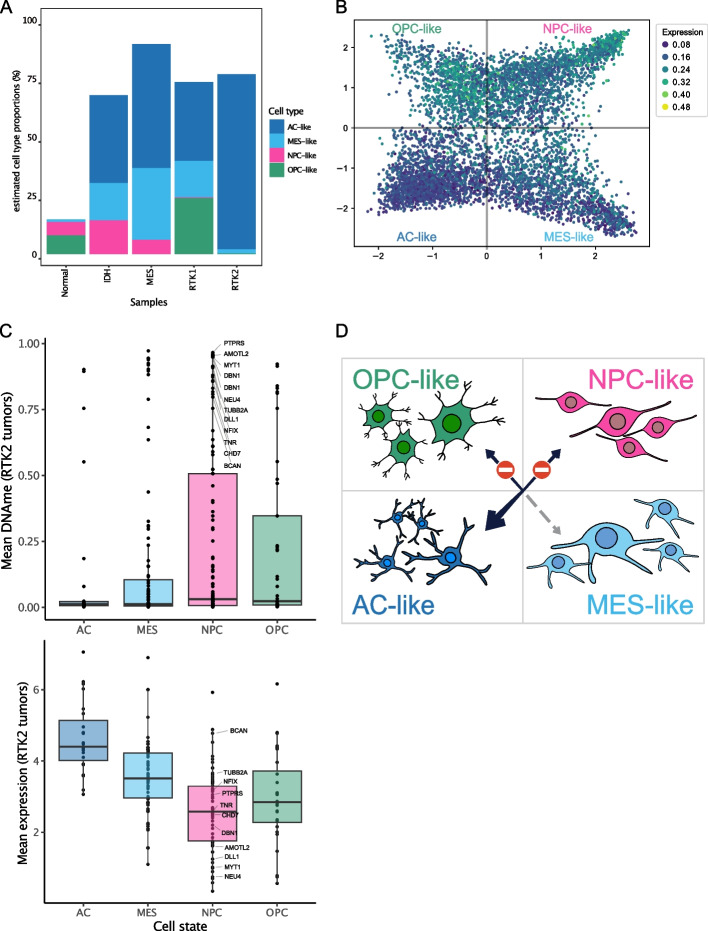
Cell state heterogeneity in glioblastoma is linked to CIMP. **A** Two-dimensional representation of the four cellular states and RTK2-CIMP expression scores of each cell. The colors indicate the expression of genes affected by RTK2-CIMP in the cells. Quadrant coordinates are based on the expression of gene modules associated with each IDH-wild-type glioblastoma cell state as published [66]. **B** Cell proportions in glioblastoma tumors as estimated by ordinary least squares deconvolution and based on the known gene modules associated with each cellular state. Each cell state is represented in color, and phenotypes are indicated on the *x*-axis. **C** *(top)* Distribution of the mean DNA methylation values of CGIs (points) found in the promoter or within the genes assigned to each cellular state module in the RTK2 subtype tumors. CGIs with a high DNA methylation level (beta above .75) in the NPC module are labeled. For readability, a label is only shown on the CGI with the maximum beta value by gene if multiple CGIs are annotated to the same gene. *(bottom) *Mean gene expression (log-transformed TPM+1) of genes from the modules in the RTK2 subtype tumors. Annotated genes found on the DNA methylation plot are labeled. **D** Proposed model of the effect of CIMP on RTK2 tumors, explaining the high proportion of cells in the AC-like state (wider arrow). Transitions into OPC- and NPC-like states might be halted by CIMP. The transition to a MES-like state appears to be possible, as its suppression is likely not linked to DNA methylation alterations

To understand how RTK2-CIMP relates to the cell-state landscape described by Neftel et al., we performed a deconvolution analysis of our bulk GBM cohort using the gene modules associated with each cell state (Fig. 5A, Additional File 1: Fig S7). Normal brain and IDH-mutated samples were also included for comparison only. We found that RTK2 tumors are depleted of NPC-like and OPC-like cell states, whereas the AC-like cell state is the most abundant, consistent with the recently proposed cell state composition of the RTK2 (classical) subtype [67]. These results indicate a strong association between RTK2-CIMP and the AC-like cell state. We hence hypothesized that the RTK2-CIMP hypermethylation leads to a repression of NPC-like and OPC-like gene programs. To confirm this, we calculated the RTK2-CIMP expression score used in the previous section for all cells of the scRNA-seq dataset from Neftel (Fig. 5B). The highest RTK2-CIMP expression score was found in NPC-like cells, together with OPC-like cells, while AC- and MES-like cells presented low expression scores. Hence, upon hypermethylation in RTK2, these OPC-/NPC-like gene programs will be silenced in RTK2, leading to a predominance of alternative cell states. Taken together, these results indicate that the expression of RTK2 CIMP target genes is mostly repressed in AC-like cells due to their hypermethylation that is driven by RTK2-CIMP.

To further confirm this link between hypermethylation and expression, we compared the expression and DNA methylation levels at the CGIs on the genes of each module (Fig. 5C). In RTK2 tumors, DNA methylation was highest for the NPC-like gene module, followed by the OPC-like module. This is also reflected by the expression of the genes from these modules, with NPC-like markers showing the lowest expression in RTK2 tumors. This finding suggested that RTK2-CIMP leads to the suppression of the NPC- and OPC-like states specifically and the establishment of the predominant AC-like state in the RTK2 subtype (Fig. 5D).

Recently, we found AC-like cell states enriched in glioblastoma patient tumors with low expression of the oligodendrocyte lineage factor driver SRY-Box Transcription Factor 10 (SOX10). Furthermore, *Sox10* suppression by genetic knockdown resulted in the transition to an AC-like cell state in mouse glioblastoma cells in vitro [68]. Therefore, we hypothesized that SOX10 knockdown might drive the observed cell-state transition through DNA hypermethylation. We tested this hypothesis by knocking down *Sox10* and analyzing CGI methylation, showing that SOX10 suppression in the mouse glioblastoma cell line mGB1 indeed resulted in RTK2-CIMP-like DNA hypermethylation (Wilcoxon test, *P* < 2.2e − 16) (Additional File 1: Fig S6A, B) and the OPC-to-AC-like cell state transition (Additional File 1: Fig S6C, D). These findings provide evidence that RTK2-CIMP is associated with the AC-like cell state in a dynamic experimental system.

## Discussion

In this work, we identified RTK2-CIMP as a new pattern of DNA hypermethylation in glioblastoma. We showed that it targets neuronal lineage genes and differs from the IDH mutant-driven CIMP of glioblastoma.

IDH-CIMP is caused by gain-of-function mutations in IDH1 and IDH2, leading to the production of the oncometabolite 2‑hydroxyglutarate, the inhibition of α‑ketoglutarate-dependent TET DNA demethylases, and consequential retention of DNA hypermethylation [15, 39, 40, 69]. This IDH mutation-dependent mechanism of gene suppression is cancer-specific and does not occur during normal development.

Here, we showed that IDH-CIMP affects CGIs broadly, while RTK2-CIMP appears to be linked to particular epigenetic signatures. RTK2-CIMP predominantly targeted CGIs with bivalent chromatin states, marked by H3K27me3 and H3K4me1. The repressive H3K27me3 mark is deposited by EZH2, a component of the polycomb repressive complex 2 (PRC2) protein complex [70]. H3K27me3 has been reported to co-occur with DNA methylation, except at CGIs, where the two marks appear mutually exclusive [24, 71]. PRC2 maintains cellular identity during normal development by silencing developmental genes [72]. Here, we compared the occupancy of histone marks and DNA methylation on RTK2-CIMP CGIs in neural progenitor and tumor cells using a Bayesian network representation to better understand the directional dynamics of histone modifications and DNA methylation. This approach, which goes beyond previous association studies, suggested that the loss of H3K27me3 at RTK2-CIMP regions could be the driving event for RTK2-CIMP hypermethylation (Additional File 1: Fig S3A, Fig. 3D).

Our findings indicate that genes affected by RTK2-CIMP are associated specifically with neuronal cells in normal neurodevelopment (Fig. 4A). Therefore, early lineage specification processes [31, 73, 74] are likely targets of RTK2-mediated repression in glioblastoma. Supporting this hypothesis, RTK2-CIMP was found to target genes essential for normal neuronal differentiation and function in neural precursors and tumors [75–78], including *NEUROG1*, *TFAP2B*, *EBF1*, and *POU4F1*. This finding was consistent with our deconvolution analysis of glioblastoma bulk RNA-seq data, which showed that neuronal cell types are absent in RTK2 tumors and that these cell types are almost exclusively associated with an astrocytic profile (AC-like), in which neural progenitor cell markers are generally expressed at low levels [66]. These results suggest that DNA hypermethylation is an essential factor determining the glioblastoma cell state. Specifically, RTK2-CIMP appears to condition glioblastoma cells to an AC-like cell state by suppressing the activity of NPC-like and OPC-like cell state genes (Fig. 5C). Notably, the downregulation of neuronal-lineage and progenitor markers did not appear to be mediated by hypermethylation of DNA or histones in the MES-like cells, suggesting that neuronal lineage silencing occurs through alternative mechanisms in these cells [79].

## Conclusions

In summary, our results suggest that, with RTK2-CIMP, glioblastoma adopted an epigenetic mechanism for cell-lineage-specific repression of normal neurodevelopment to regulate tumor cell-state transitions and phenotypes. Taken together with previous research on the variability of active enhancer regions [46], this work implies DNA methylation as a critical epigenetic element involved in cell and tumor heterogeneity in glioblastoma.

## Methods

### Sequencing data processing

WGBS, ChIP-seq and RNA-seq data from glioblastoma tumors were processed as previously described [46]. Subtype classification previously performed was also used [35].

The ChIP-seq histone modification for NP and HPSC and methylation for HPSC datasets were obtained from ENCODE as processed BigWig files [52, 80]. Processed NP data for methylation (WGBS) were obtained from the GEO dataset GSE156723 [51].

Methylation data for AML and normal blood samples were obtained from the TCGA (TCGA-AML) and GEO (GSE51388) databases, respectively. Both sets of data are based on the Illumina 450 K methylation platform. The methylation level of each CGI was defined by the average beta values of all the CpGs located on it.

### Definition of CIMP-CGIs

CGIs affected by CIMP were defined on the basis of published thresholds [5]. CIMP-CGIs that display hypermethylation relative to CIMP-negative tumors (in glioblastoma, MES, and RTK1; when |methylation beta difference|> 0.2), low methylation in the normal brain or blood (when methylation beta < 0.75), and statistical significance upon subtype-specific testing (when Kruskal‒Wallis test, adj. *p* < 0.001) were considered CIMP-CGIs.

### Cox-proportional hazard analysis

RTK2-CIMP methylation score was computed on the RTK2 subtype samples of the TCGA GBM cohort by averaging the methylation values of the RTK2-CIMP CGIs. Clinical information about the samples was retrieved through the RTCGA R package (1.32.0). A Cox-proportional hazard analysis was then performed with the survival R package (3.7.0) using the RTK2-CIMP methylation score as continuous predictor, yielding a significant association between stronger hypermethylation and increased survival (*n* = 58, Coefficient = − 4.51, *p*-value = 0.0245, 95% confidence interval for hazard ratio: 0.0002 < HR < 0.56).

### Validation of RTK2-CIMP in glioblastoma samples from TCGA

Processed 450K array DNA methylation data for IDH-wild-type glioblastoma were obtained from the TCGA (*n* = 131). The samples were classified into existing subtypes using the same DNA methylation classifier that was previously applied to the WGBS data [46]. CpG beta values overlapped with CGIs, and mean beta values for the CGIs were used in the DNA methylation assessment. The overlap with IDH-CIMP was excluded from the visualizations shown. A random sample of equal size including only non-CIMP CGIs was used for the comparisons.

### Knockdown of Sox10 in the mGB1 cell line

In mGB1 cells, Sox10 was stably knocked down using short hairpin RNA (shRNA) as previously described (Wu et al., 2020). Briefly, nonoverlapping target sequences of shRNA sequences (sh1: TRCN0000018985; sh2: TRCN0000244290) were cloned and inserted into pLKO.1-puro (Sigma, SHG002). For the delivery of shRNAs, lentiviruses of pLKO.1-non target control, sh1, and sh2 were produced and transduced into mGB1 cells. Transduced cells were selected with 1 µg/ml puromycin for 48 h, and the K.D. efficiency was checked by both qPCR and Western blot.

### DNA methylation array profiling

Sox10 knockdown and control cells were profiled using Infinium MouseMethylation285k BeadChip arrays in the Microarray Core Facility at DKFZ, and the data were analyzed using the minfi (1.24.0)^63^ and conumee (1.3.0)^64^ Bioconductor packages. In brief, > 500 ng of DNA from snap-frozen samples was used as input material. minfi was used to extract raw signal intensities from IDAT files, and both color channels were corrected for background and dye bias. Beta values were calculated using an offset of 100.

### Analysis of DNA methylation array

Beta values for the CpGs generated by the core facility were used for downstream analysis. Replicates were averaged by group (control or *Sox10* knockdown). Probes below the significance threshold (*p*-value > 0.05) and CHG probes were excluded from downstream analysis. RTK2-CIMP locations (including overlaps) were lifted from the human assembly hg19 to the mm10 mouse genome. This conversion resulted in the loss of 205 CGIs.

CpGs overlapping the liftovered RTK2-CIMP regions were referred to as RTK2-CIMP and a comparable sample of CpGs was used for comparison. CpGs were considered to be altered between the control and knockdown groups if the log2-fold change was greater than 0.01 (gain) or less than − 0.01 (loss).

### CGI-based NMF analysis

All CGIs (*n* = 26,268) were used for the NMF analysis using the ButchR package (v1.0) [54]. This approach was applied to the combined CGI-averaged outputs of *multiBigwigSummary* obtained from the ChIP-Seq and WGBS data on both the glioblastoma and NP datasets. NMF computation was performed with 10^4^ iterations, 20 initializations, and rank factorization from 3 to 10. The final factorization rank selected, according to observation and quality metrics, was 4.

CGIs are assigned to each signature using the *SignatureSpecificFeatures* function from ButchR for signature-uniquely assigned CGIs [54]. If the function assigns CGIs to more than one signature, CGIs are assigned to the signature for which their W-matrix normalized exposure value is ≥ 0.8. All CGIs are assigned to at least one signature. If the normalized exposure value of a given CGI is ≥ 0.8 in more than one signature, CGIs are considered multiply assigned.

### Multi-tissue assessment of H3K27me3 enrichment

To quantify the H3K27me3 levels on the A/RTK2-CIMP CGIs in hESCs, we used H3K27me3 ChIP-seq-processed signals from the H9 cell line (ENCFF018IRK) as published in ENCODE [52, 80]. MultiBigSummary was used to compute the average fold change over the control (input) over the target CGIs [81].

### Random forest classification

To distinguish between the two classes of CIMPs (RTK2-/A-CIMP vs IDH-CIMP) in both glioblastoma and AML patients, we generated an RF classification model with 9 predictors. Overlap between the A-CIMP group and IDH-CIMP group were excluded from the classification. Training included 2724 CGIs (70%), and testing included 1166 CGIs (30%). The training was performed with 2000 trees, cross-validation (tenfold repeated 5 times), and downsampling using the caret R package.

### Bayesian network construction

Bayesian networks for RTK2-CIMP and IDH-CIMP were generated using the R package *bnlearn* [82]. Networks were trained with bootstrapping using epigenomic data from the affected CGIs on NPs over the 7 epigenetic features.

### Rank-based comparison between NPs and RTK2 using H3K27me3 and DNA methylation

To accurately compare CGIs between NPs and RTK2 tumors, we ranked each CGI according to its enrichment in H3K27me3 and DNA methylation. Ties are solved using permutation with increasing values at each index set of ties (or “first”). The rank difference was then calculated between RTK2 and the NPs (i.e., the CGI with the highest DNA methylation value in RTK2 was ranked as 1, but if it ranked as 1000 in the NPs, its rank difference would be − 999).

### Brain cell population comparisons

RTK2-CIMP and IDH-CIMP targets were compared with two sets of brain markers [61, 62]. For the *Couturier* set, 49 genes were selected according to their *q*-value (≤ 0.01) and log2-fold change (> 1). Subpopulations of neurons, neuron progenitors, and radial glia were collapsed into *neurons/interneurons, neuronal progenitors*, and *radial glia*, respectively. The *McKenzie* set (*n* = 66) was used for comparison as published. The list of genes in each set is available in Additional File 2: Table S1.

### Single-cell RNA-seq analysis

In the normal brain and development analysis, we used published brain expression data to assess the differences between the impact of RTK2-CIMP and IDH-CIMP on cellular development. We used a published brain development dataset [63] and the Allen Brain Atlas [64] to determine CIMP expression scores in developing and adult brains, respectively. Cell clustering was used for visualization as published. CIMP expression scores were calculated using CIMP-affected genes at the promoter using the *AddModuleScore* function in Seurat (v4.1.0). For the clustering and log2-fold change (RTK2-/IDH-CIMP) representation, negative CIMP expression scores were converted to 0 to allow log2-fold change calculation.

In AML/blood, scRNA-seq data of human developmental hematopoiesis were used to explore the expression of CIMP-targeting genes involved in hematopoiesis [83]. An H5ad file was obtained for the processed scRNA-seq data, and Scanpy (v.1.9.1) in Python was used to score the A- and IDH-CIMP-targeting genes and perform uniform manifold approximation and projection (UMAP)*.*

In glioblastoma, scRNA-seq data (Smartseq2) from IDH-wild-type tumors were obtained from the Broad Institute Single-Cell Portal [66]. The publicly available coordinates were used for the two-dimensional representation of the cellular state. Cells were scored according to the expression of RTK2-CIMP-affected genes. For consistency of visualization, the NPC and MES modules were merged from the NPC1 and NPC2 modules and the MES1 and MES2 modules, respectively.

### Deconvolution of bulk tissue

The *granulator* R package was used to estimate the composition of different cell states in bulk RNA-seq data in glioblastoma using gene module expression [66]. The ordinary least squares deconvolution method was implemented. For inputs, reference expression and gene modules for each cell state were obtained as publicly available [66]. For visualization and further interpretation, the gene modules MES1 and MES2 and NPC1 and NPC2 were combined into the MES and NPC gene modules, respectively. Results obtained using alternative algorithms are consistent with the ols results and displayed in Additional File 1: Fig S7.

### Single-cell DNA methylation of IDH-wild-type tumors

The data were provided as an already processed Seurat object by the authors of the original publication [47]. Identification of RTK2 and non-RTK2 samples was based on cell state fractions determined in the original publication, *EGFR* amplification status, and gene expression (not included here). The category “Others” was generated to group cycling cells and is sometimes omitted to simplify representation. Due to the low coverage of the dataset, DNA methylation scores were estimated using the ratio of methylated to unmethylated covered CpGs located within the RTK2-CIMP region for each cell state. The ratio was calculated within each CpG island present.

## Supplementary Information

Additional File 1: Supplementary Figure S1 – S7.

Additional File 2: Supplementary Table S1.

## Data Availability

DNA methylation, gene expression, and ChIP-seq (on H3K27ac, H3K4me1/3, H3K36me3, H3K27me3, and H3K9me3) data analyzed in this study were obtained from the Gene Expression Omnibus (GEO) database under accession code GSE121723 [84]. Histone modification ChIP-seq (targeting H3K27ac, H3K4me1/3, H3K36me3, H3K27me3, and H3K9me3) and DNA methylation data for NPs originating from H9 were obtained from ENCODE (reference epigenome ENCSR372BDU) and GSE156723 datasets, respectively [85, 86]. The RRBS data from [47] can be obtained from the authors of the original publication upon request. The mouse DNA methylation data on Sox10 knockdown and control is available from Gene Expression Omnibus (GEO) database under accession code GSE296674 [87].
