# Supplementary material for: A new IDH-independent hypermethylation phenotype is associated with astrocyte-like cell state in glioblastoma
Source: Genome Biol. 2025 Jul 3;26:192. doi: 10.1186/s13059-025-03670-y (PMC12225510; doi:10.1186/s13059-025-03670-y)
Supplement: Supplementary file 1 — Additional File 1: Supplementary Figure S1 – S7. [file 13059_2025_3670_MOESM1_ESM.docx]

Figure S1


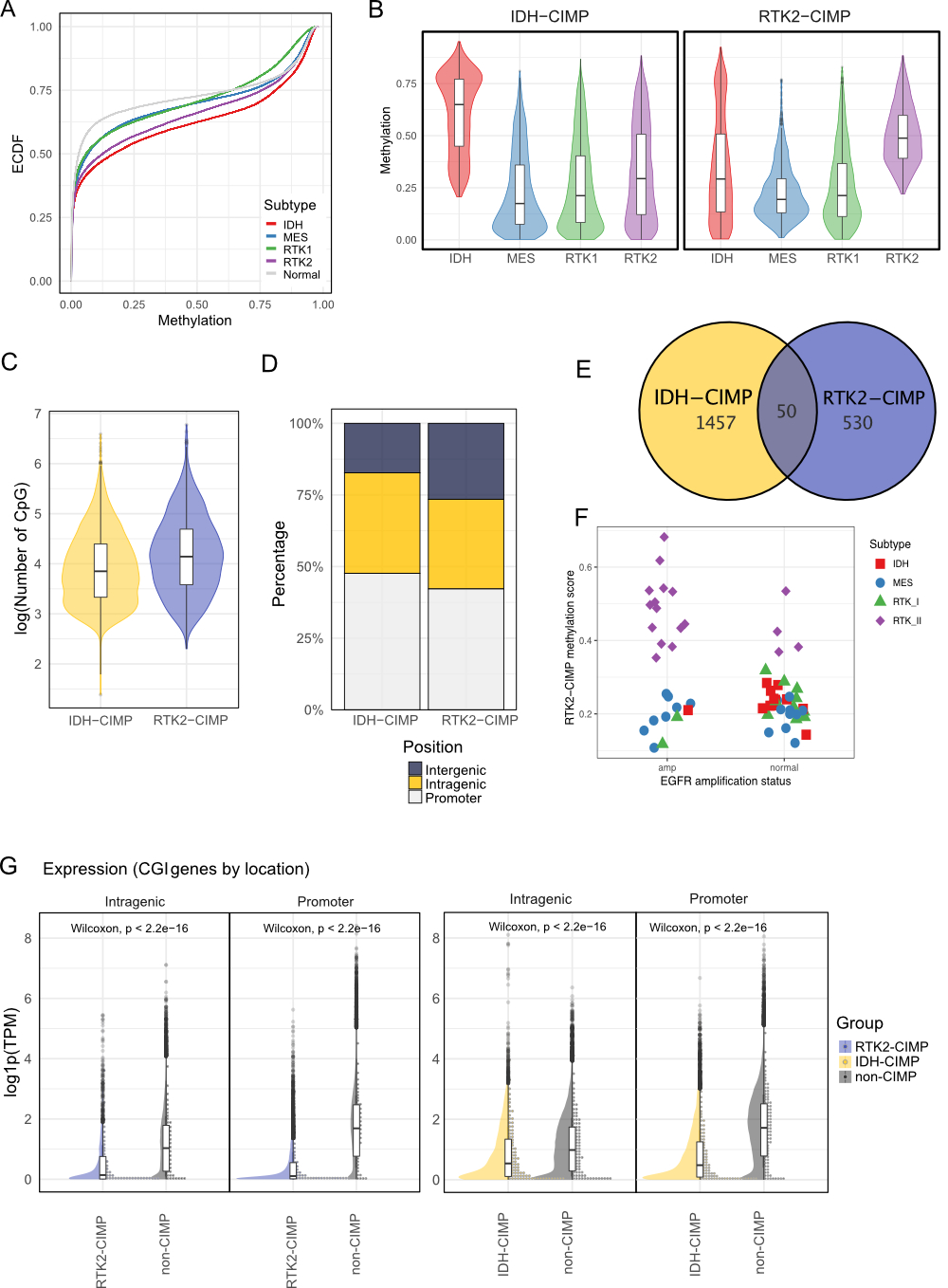


**Figure S1**

A) Empirical cumulative distribution function curves for DNA methylation level on all CGIs in the different groups (GBM subtypes are represented in their respective color code). DNA methylation in the normal brain is represented in light gray.

B) Distribution of DNA methylation (in beta-values on y-axis) of IDH- and RTK2-CIMP CGIs for the different GBM subtypes. Subtypes are indicated in the x-axis and represented in color.

C) Number (log-transformed) of detected CpGs by CGIs assigned to either IDH- (yellow) or RTK2-CIMP (blue).

D) Location annotation (in percentage) of CGIs assigned to either IDH- or RTK2-CIMP in relation with genes.

E) Number of genes from the RTK2- and IDH-CIMP groups and of their overlap.

F) Average methylation score over all RTK2-CIMP regions for all samples (y-axis), stratified by EGFR amplification status (x-axis).

G) Expression level (log-transformed TPM+1) of genes affected by CGIs assigned to either IDH- (yellow) or RTK2-CIMP (blue) faceted according to location. Genes annotated to other CGIs (non-CIMP) are shown in gray. Wilcoxon signed-rank test (two-sided) p-value is shown.

Figure S2


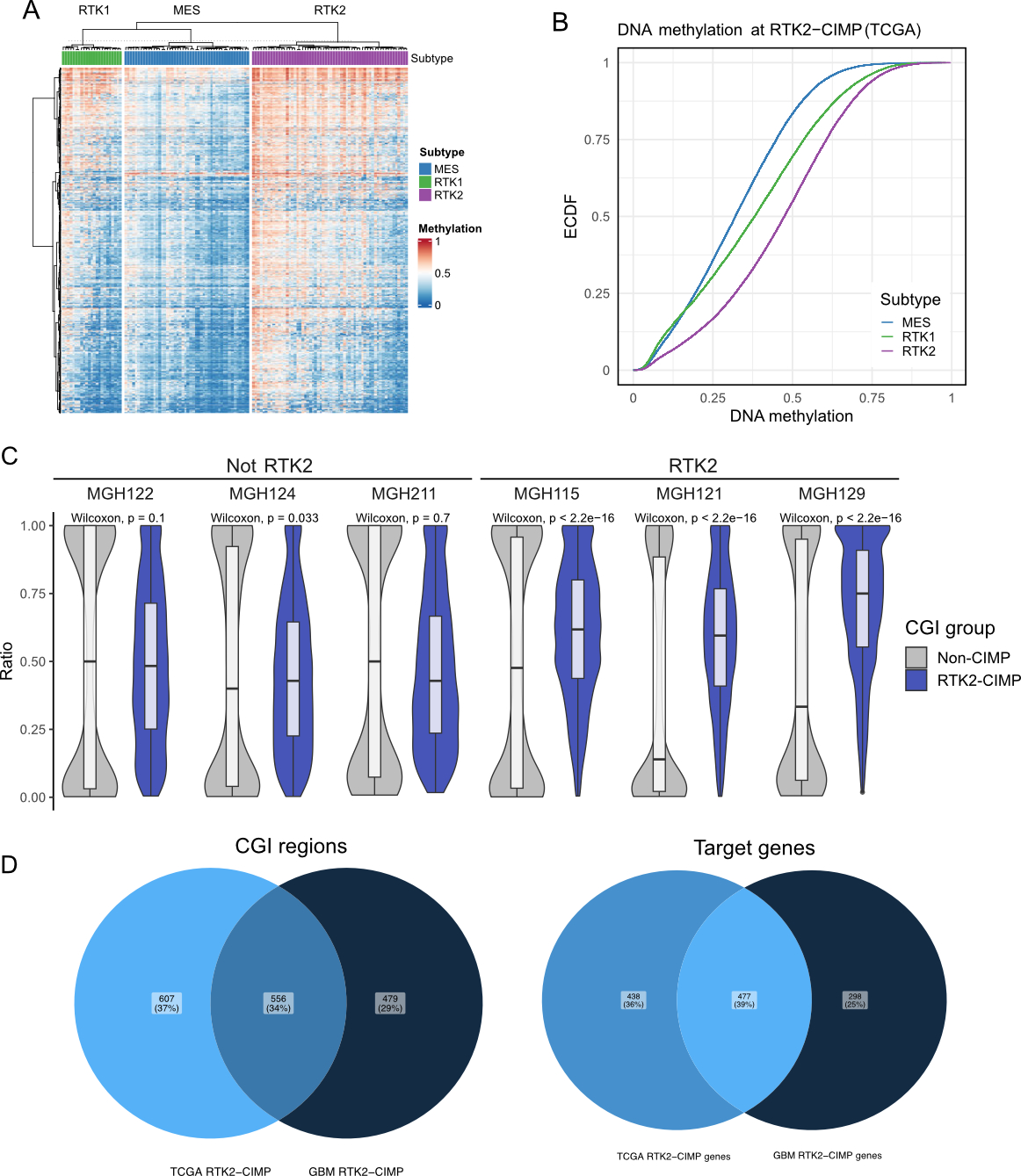


**Figure S2**

1. Heatmap representing the DNA methylation at RTK2-CIMP in the TCGA cohort. Samples are annotated into the 3 subtypes (top).
2. Comparison between RTK2-CIMP in the three subtypes assigned to the IDH-wild type glioblastoma samples from TCGA.
3. Fraction of methylated CpGs in the single-cell dataset from Chaligne et al. in non-RTK2 (3 samples on the left) and RTK2 (3 samples on the right) samples, in non-CIMP vs. RTK2-CIMP-CGI regions
4. Comparison of RTK2-CIMP CGI regions defined from the TCGA cohort, compared to the GBM cohort of this study (left); Comparison of the number of target genes for the sets of RTK2-CIMP CGIs defined in the TCGA and GBM cohort (right).

Figure S3


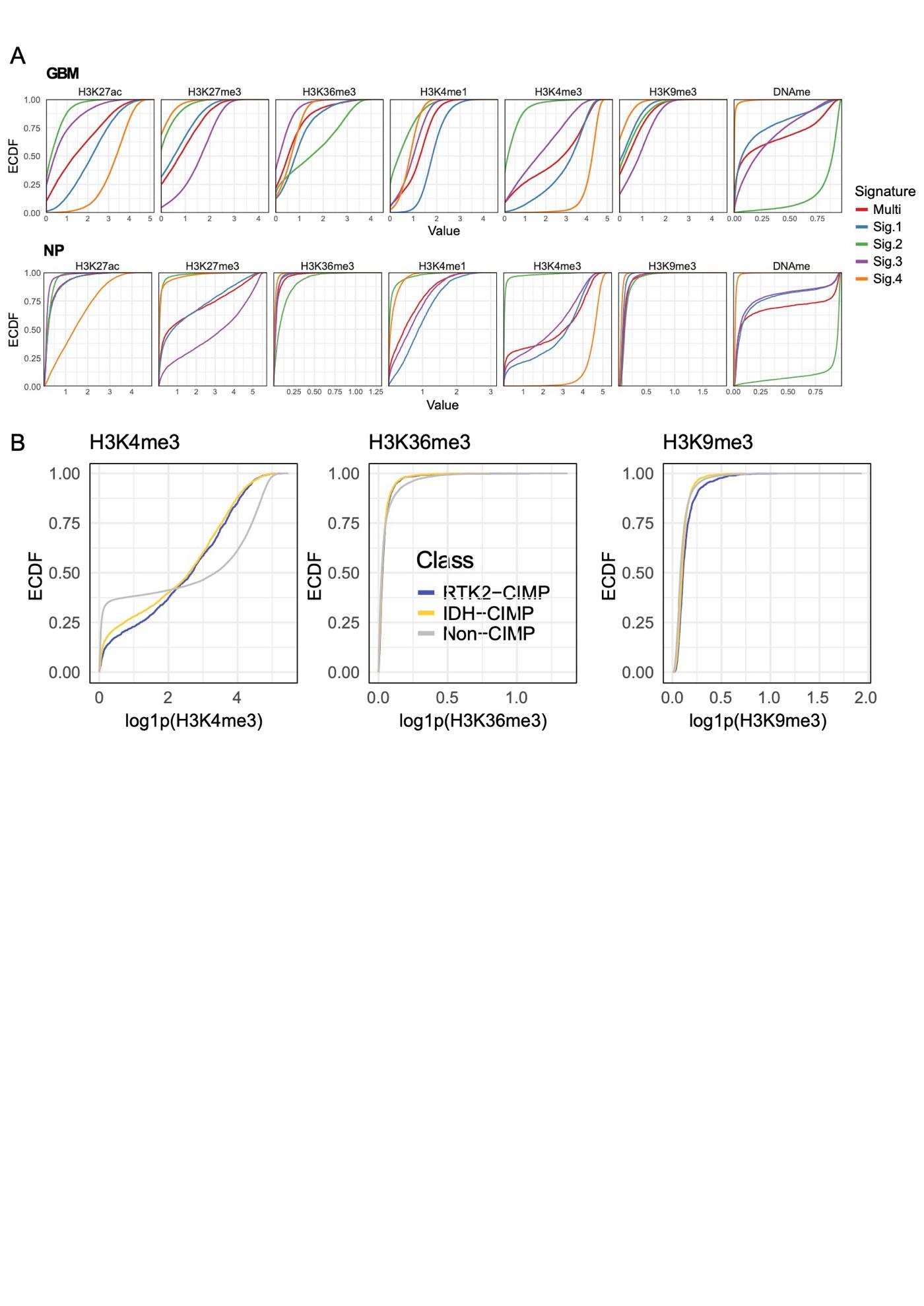


**Figure S3**

A) Empirical cumulative distribution function curves of each CGI signature on the 7 distinct epigenetic modifications for both GBM *(top)* and NPs *(bottom)*. Each square panel represents one epigenetic modification and its level in each NMF-derived CGI signature (line colors). GBM plots are composed of the combined set of all 4 subtypes.

B) Enrichment in different histone modifications on the NPs by CIMP group. Empirical cumulative distribution function curves represent the CGIs assigned to either IDH- (yellow line), RTK2-CIMP (blue line), or Non-CIMP (gray line).

Figure S4


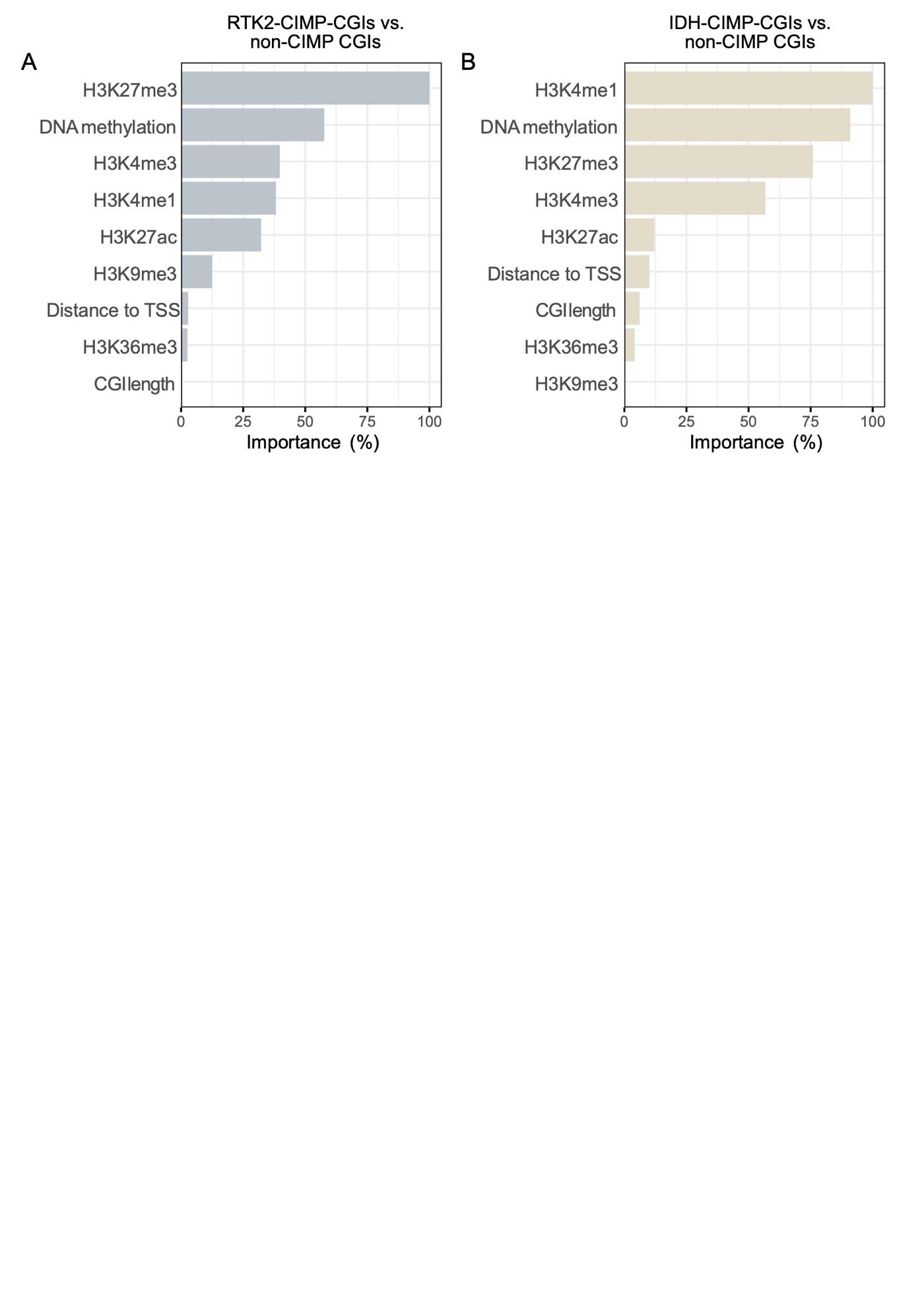


**Figure S4**

A) Feature importance (in percentage in the x-axis) obtained on the RF model trained to classify RTK2-CIMP and non-CIMP CGIs according to their respective CGI features (y-axis).

B) Feature importance (in percentage in the x-axis) obtained on the RF model trained to classify IDH-CIMP and non-CIMP CGIs according to their respective CGI features (y-axis).

Figure S5


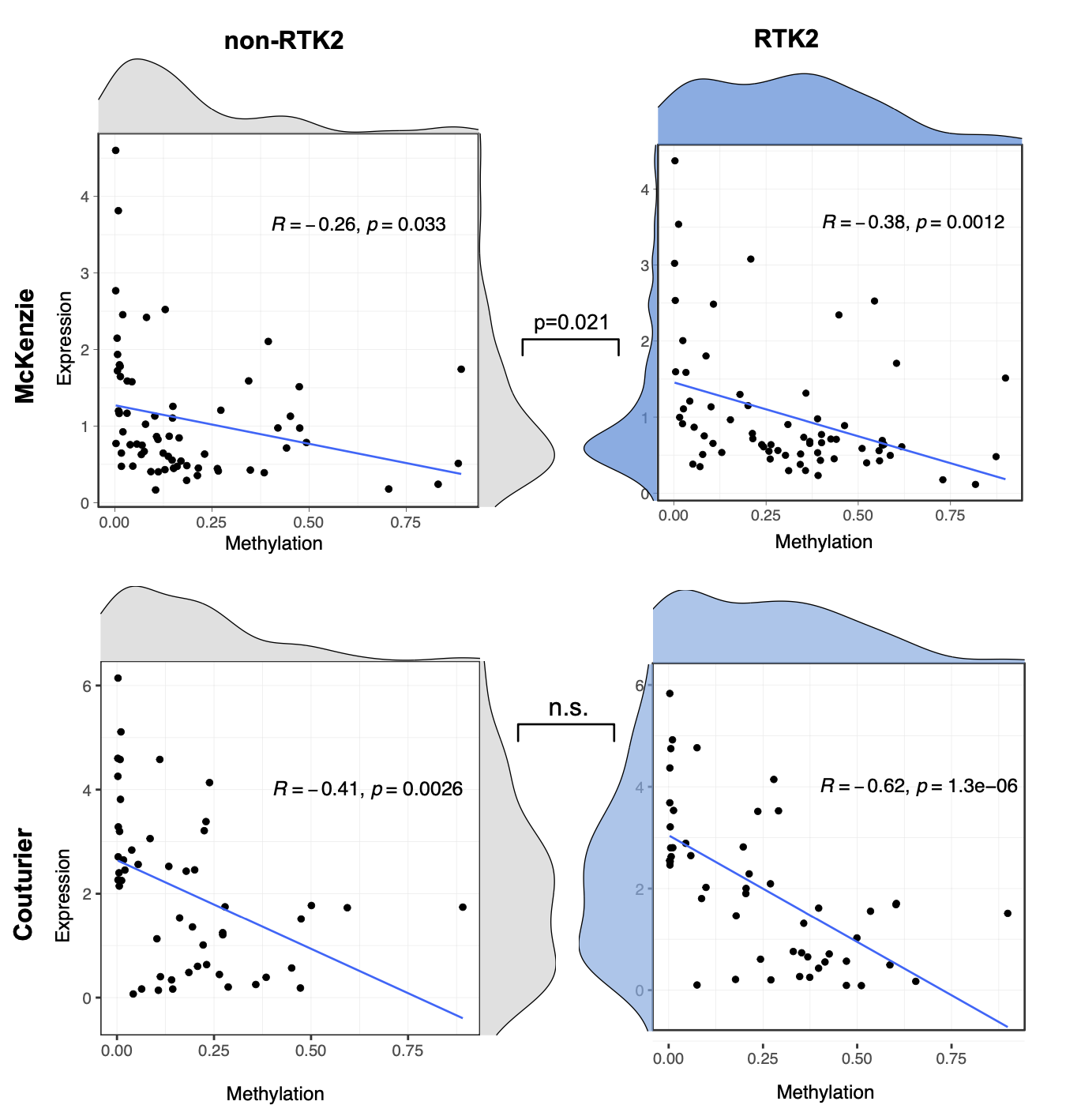


**Figure S5**: Correlation between the expression of the neuronal marker genes (y-axis) and the methylation of the associated CGI (x-axis) for marker genes from the McKenzie set (top row) or Couturier set (bottom row). Each dot represent one gene, and the x- and y-values are averaged over the samples from the RTK2 set (right column) or non-RTK2 (i.e. MES and RTK1, left column). The expression difference between RTK2 and non-RTK2 is significant for McKenzie, but not for Couturier.

Fig S6


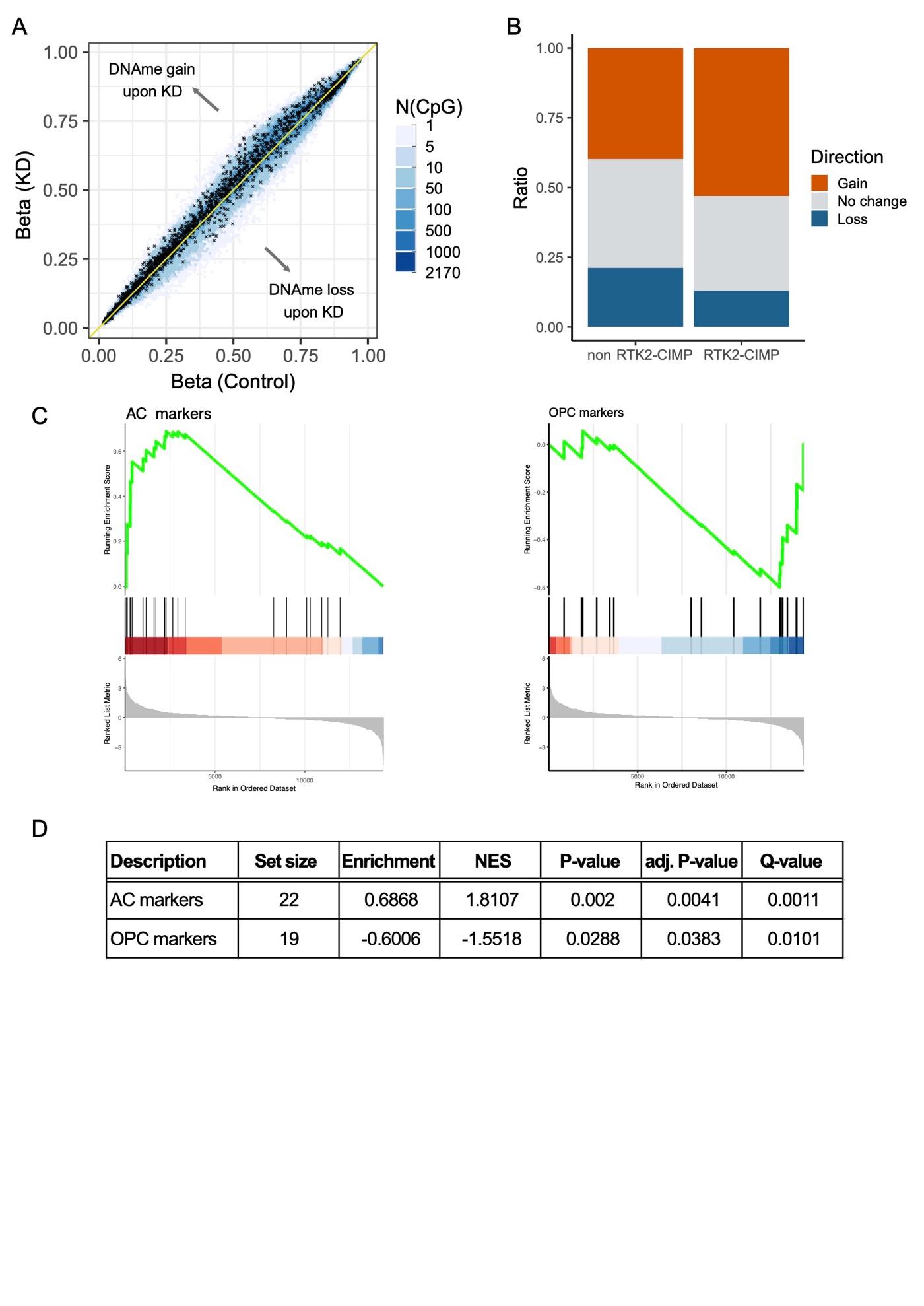


**Figure S6**

A) DNA methylation (beta values) between control (NT, x-axis) and SOX10 knockdown (y-axis) samples for all CpGs (blue dots) and CpGs overlapping RTK2-CIMP-CGI regions (black crosses). B) Proportion of CpGs showing methylation gains, losses or no change in DNA methylation for non-RTK2-CIMP CGIs (left) or RTK2-CIMP-CGIs (right). C) GSEA enrichment of AC markers (left) or OPC-markers (right) in the list of genes ranked by log-fold-change gene expression comparing KD vs control condition. D) GSEA enrichment statistics.

Figure S7


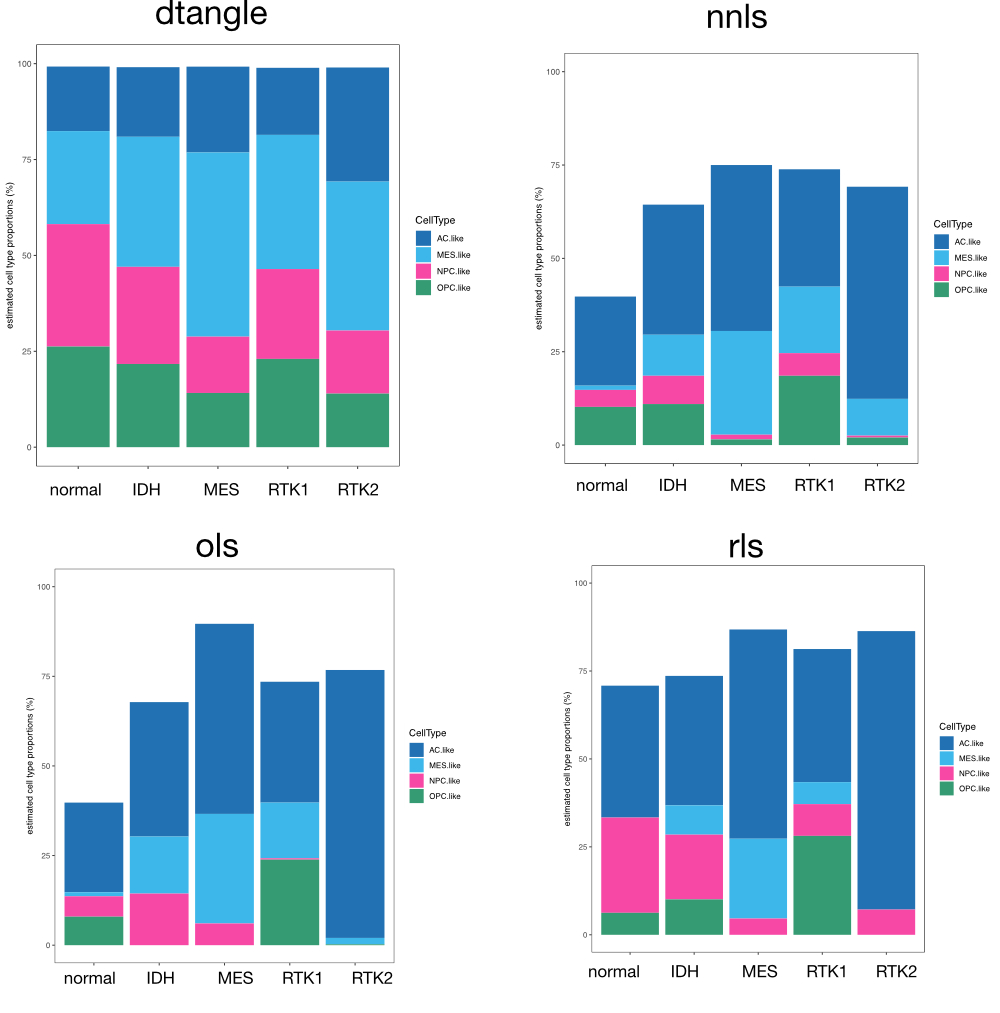


**Figure S7**: Deconvolution results obtained using the granulator package with different deconvolution algorithms implemented in the package. Missing proportions to reach 100% represent unassigned cells, i.e. could represent normal cells, immune cells or unidentified tumor cells.
